# Supplementary material for: Non-enzymatic ABHD6 interacts with Akt-FoxO1 axis to regulate selective hepatic insulin resistance
Source: bioRxiv. 2026 Feb 13:2026.02.11.705361. Preprint. [Version 1] doi: 10.64898/2026.02.11.705361 (PMC12918827; doi:10.64898/2026.02.11.705361)
Supplement: Supplement 1 [file NIHPP2026.02.11.705361v1-supplement-1.pdf]

bioRxiv preprint doi: https://doi.org/10.64898/2026.02.11.705361; this version posted February 14, 2026. The copyright holder has placed this preprint (which was not certified by peer review) in the Public Domain. It is no longer restricted by copyright. Anyone can legally share, reuse, remix, or adapt this material for any purpose without crediting the original authors.

Supplementary information

Non-enzymatic ABHD6 interacts with Akt-FoxO1 axis to regulate selective hepatic insulin resistance

A. Supplementary Figures.
   1. High fat diet induces liver insulin resistance.
   2. Deletion of ABHD6 in liver protects the mice from MASLD and liver fibrosis.
   3. Overexpression of ABHD6 in the liver drives insulin resistance through a lipid-independent mechanism.
   4. ABHD6 acts as a BMP hydrolase yet modulates insulin signaling independently of BMP.
   5. ABHD6 is a pivotal regulator of FoxO signaling.
   6. ABHD6 manipulation only affects Akt-Foxo1/3a-mediated gluconeogenesis, without affecting Akt-mediated lipogenesis.
   7. ABHD6 regulates Akt-Foxo1/3a phosphorylation and interaction, leading to altered gluconeogenesis.
   8. ABHD6 regulates insulin sensitivity in a FoxO1-dependent manner.

B. Supplementary Table
   1. Primers used in the study.
   2. Antibodies used in the study.
   3. DEG pathway in response to ABHD6 manipulation.

bioRxiv preprint doi: https://doi.org/10.64898/2026.02.11.705361; this version posted February 14, 2026. The copyright holder has placed this preprint (which was not certified by peer review) in the Public Domain. It is no longer restricted by copyright. Anyone can legally share, reuse, remix, or adapt this material for any purpose without crediting the original authors.

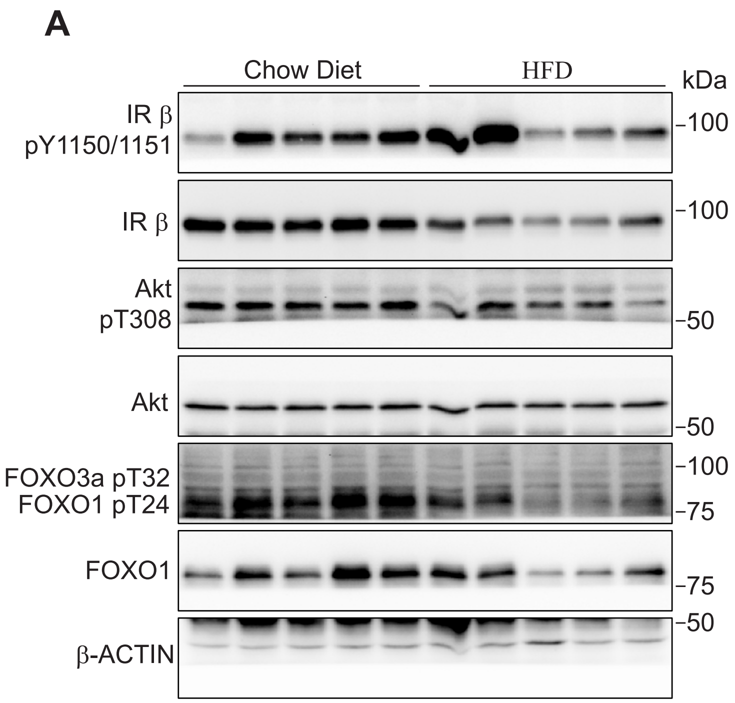

Sup Figure 1: High fat diet induces liver insulin resistance. (A) Effects of high fat diet on hepatic insulin signaling by western blots.

bioRxiv preprint doi: https://doi.org/10.64898/2026.02.11.705361; this version posted February 14, 2026. The copyright holder has placed this preprint (which was not certified by peer review) in the Public Domain. It is no longer restricted by copyright. Anyone can legally share, reuse, remix, or adapt this material for any purpose without crediting the original authors.

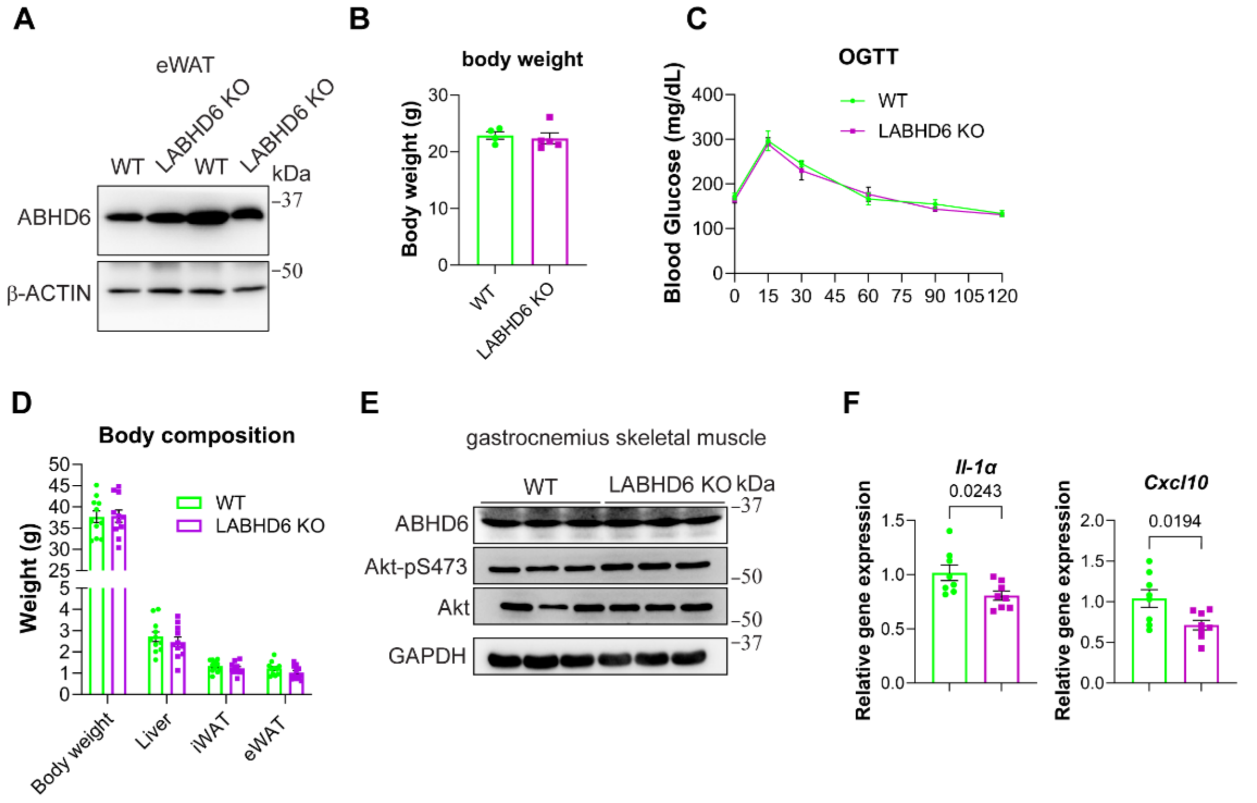

Sup Figure 2. Deletion of ABHD6 in liver protects the mice from MASLD and liver fibrosis. (A) ABHD6 protein levels in adipose tissue. (B) Body weight of WT and LABDH6 KO mice under chow diet. (C) Blood glucose levels during glucose tolerance test (GTT) (n=5 per group). (D) Body composition. (E) Immunoblot of ABHD6 and Akt activation in the gastrocnemius skeletal muscle of LABHD6 KO mice. Mice were deprived of food overnight and intraperitoneal injected with 0.25 U/kg insulin. Gastrocnemius skeletal muscle was harvested 5 minutes after insulin injection. (F) Expression of Il-1a and Cxcl10. Data are displayed as mean ± SEM and analyzed by Student's *t* test. p-values of respective comparisons are provided

bioRxiv preprint doi: https://doi.org/10.64898/2026.02.11.705361; this version posted February 14, 2026. The copyright holder has placed this preprint (which was not certified by peer review) in the Public Domain. It is no longer restricted by copyright. Anyone can legally share, reuse, remix, or adapt this material for any purpose without crediting the original authors.

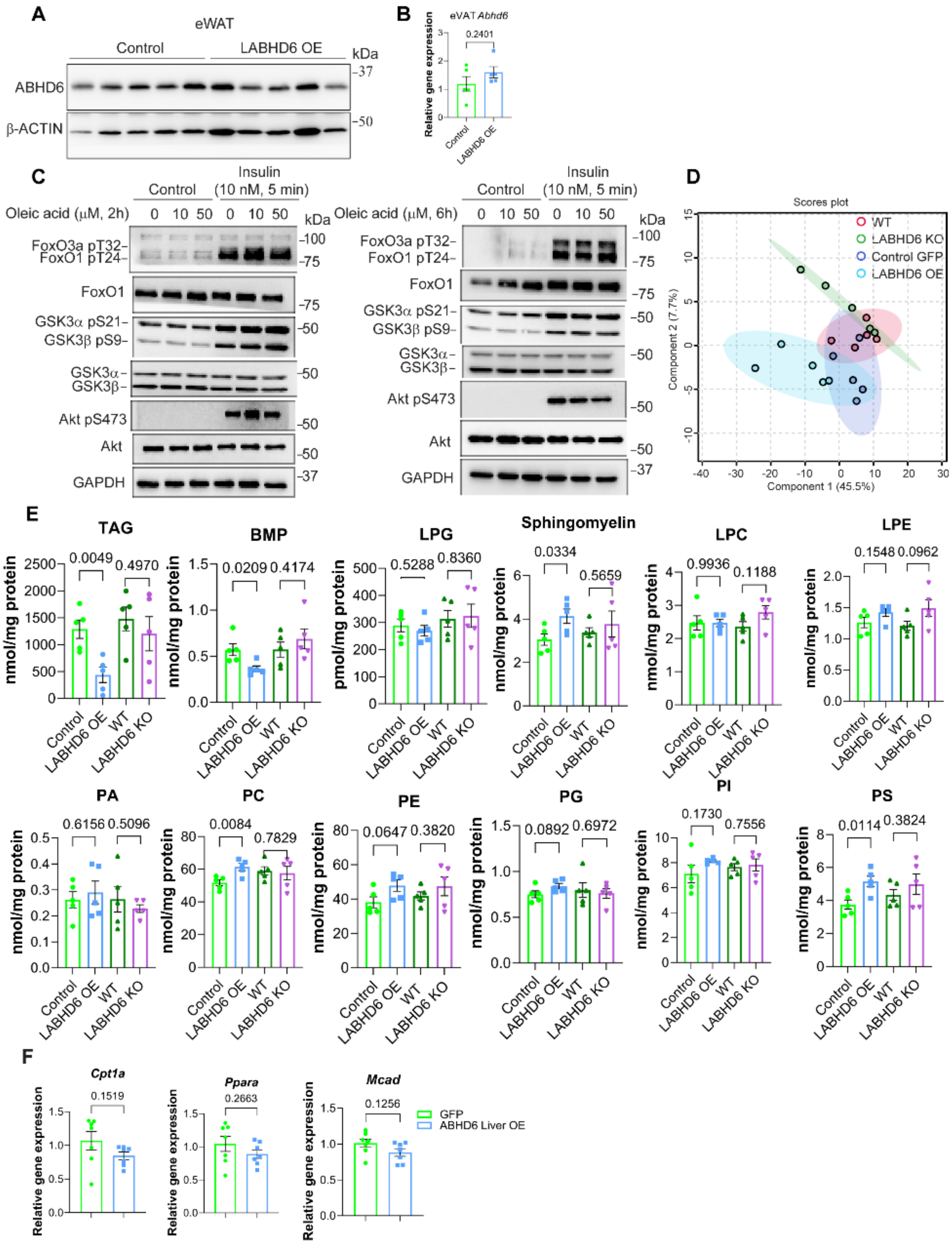

bioRxiv preprint doi: https://doi.org/10.64898/2026.02.11.705361; this version posted February 14, 2026. The copyright holder has placed this preprint (which was not certified by peer review) in the Public Domain. It is no longer restricted by copyright. Anyone can legally share, reuse, remix, or adapt this material for any purpose without crediting the original authors.

Sup Figure 3. Overexpression of ABHD6 in the liver drives insulin resistance through a lipid-independent mechanism. (A) ABHD6 protein levels in adipose tissue. (B) Gene expression of *Abhd6* in adipose tissue. (C) Effects of exogenous 1-OG administration on hepatic insulin signaling. (D) partial least squares discriminant analysis (PLS-DA). (E) Overview of the change of lipid classes in response to ABHD6 deletion and overexpression. (F) Gene expression of Cpt1a, Ppara, and Mcad. (B, E-F)Data are displayed as mean ± SEM and analyzed by Student's *t* test. p-values of respective comparisons are provided.

bioRxiv preprint doi: https://doi.org/10.64898/2026.02.11.705361; this version posted February 14, 2026. The copyright holder has placed this preprint (which was not certified by peer review) in the Public Domain. It is no longer restricted by copyright. Anyone can legally share, reuse, remix, or adapt this material for any purpose without crediting the original authors.

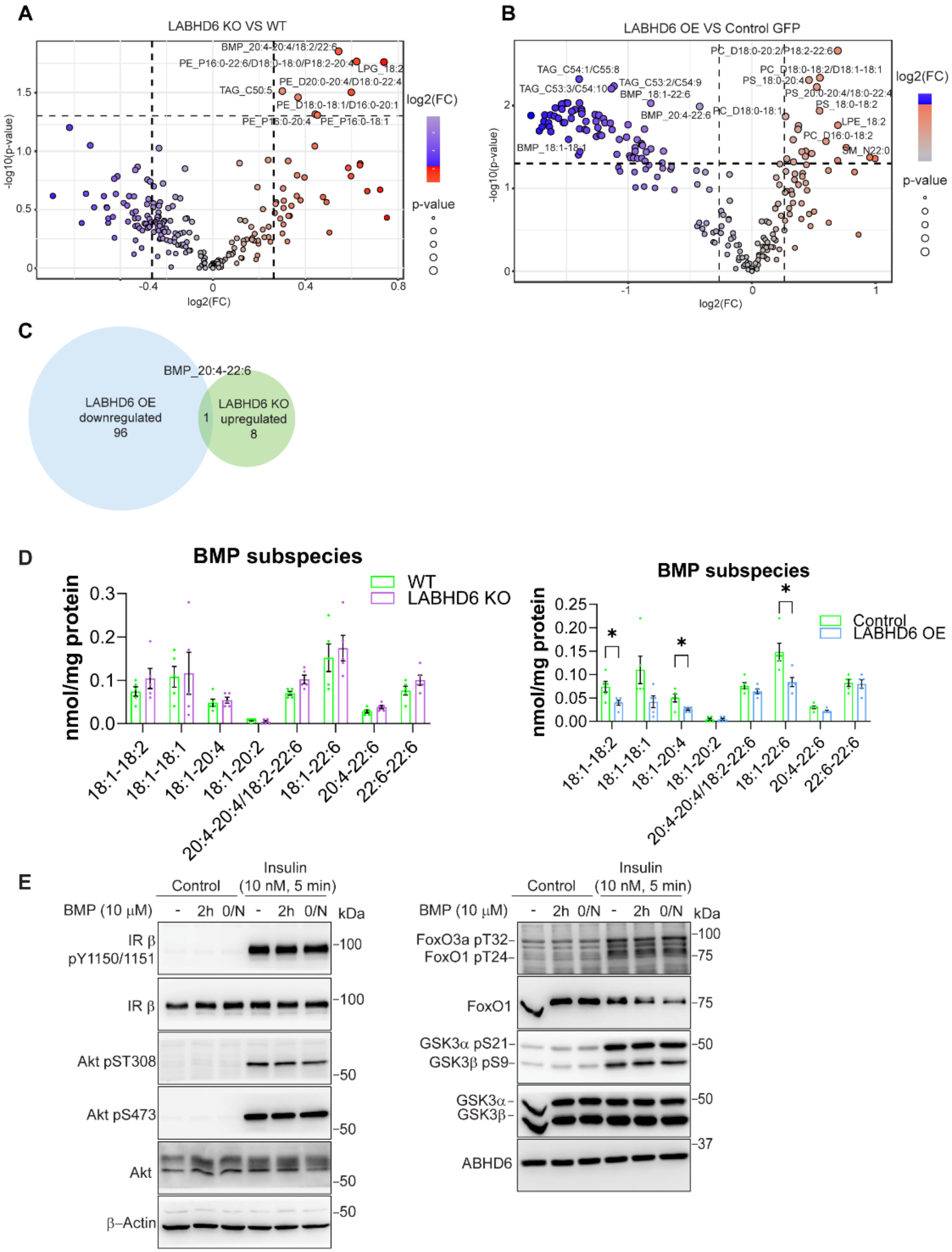

bioRxiv preprint doi: https://doi.org/10.64898/2026.02.11.705361; this version posted February 14, 2026. The copyright holder has placed this preprint (which was not certified by peer review) in the Public Domain. It is no longer restricted by copyright. Anyone can legally share, reuse, remix, or adapt this material for any purpose without crediting the original authors.

Sup Figure 4. ABHD6 acts as a BMP hydrolase yet modulates insulin signaling independently of BMP. Livers from HFD-LABHD6 KO and HFD-LABHD6 OE mice (n = 5 per group) were used for lipidomic analysis. (A and B) Volcano plots of lipid species. (C) Venn diagram of differentially abundant lipid species. (D) Total BMP subspecies in livers of LABHD6 KO and OE mice. (E) Effects of exogenous BMP administration on hepatic insulin signaling. Data are displayed as mean ± SEM and analyzed by Student's *t* test. $^{*}p < 0.05$

bioRxiv preprint doi: https://doi.org/10.64898/2026.02.11.705361; this version posted February 14, 2026. The copyright holder has placed this preprint (which was not certified by peer review) in the Public Domain. It is no longer restricted by copyright. Anyone can legally share, reuse, remix, or adapt this material for any purpose without crediting the original authors.

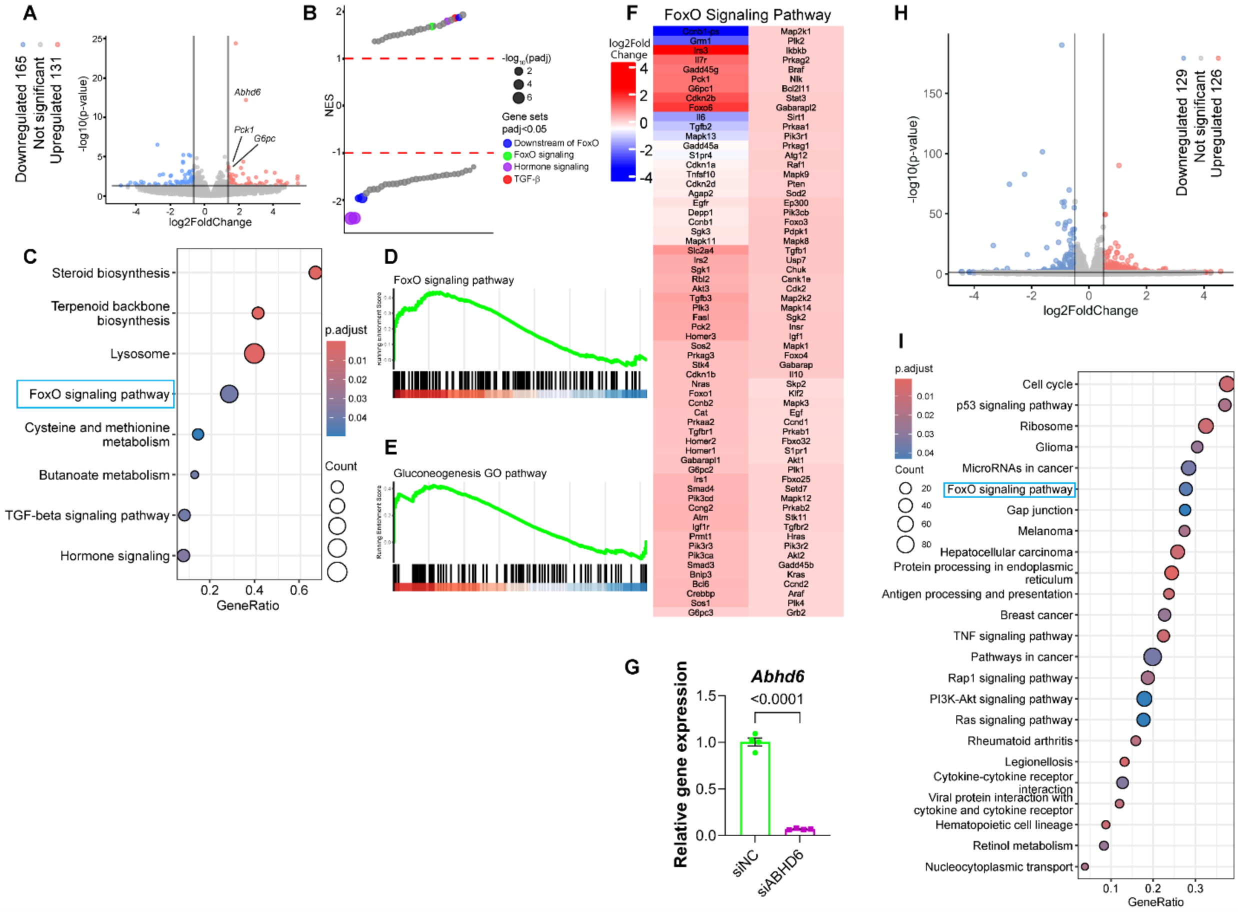

Sup Figure 5. ABHD6 is a pivotal regulator of FoxO signaling.

(A-F) 8-week-old male wildtype mice were injected with 1×10[11] genome copies AAV8 TBG-eGFP or TBG-*Abhd6* (n = 3 per group). 1 week post-injection, the mice were fed a high-fat diet (HFD) for 12 weeks. Bulk RNA sequencing was performed on livers. **(A)** Volcano plot of gene expression. **(B)** Upregulated and downregulated pathways. **(C)** Top ranked enriched pathways. **(D and E)** Gene Set Enrichment Analysis (GSEA) against ‘FoxO signaling’ and ‘Gluconeogenesis pathway’ sets. **(F)** Expression of genes in ‘FoxO signaling’ set. (G-I) Bulk RNA sequencing was performed in primary hepatocytes under conditions of ABHD6 knockdown by siRNA. (G) Verification of ABHD6 knockdown by

bioRxiv preprint doi: https://doi.org/10.64898/2026.02.11.705361; this version posted February 14, 2026. The copyright holder has placed this preprint (which was not certified by peer review) in the Public Domain. It is no longer restricted by copyright. Anyone can legally share, reuse, remix, or adapt this material for any purpose without crediting the original authors.

siRNA. (H) Total changed genes in response to ABHD6 knockdown in primary hepatocytes. (I) Top ranked pathways that affected by ABHD6.

bioRxiv preprint doi: https://doi.org/10.64898/2026.02.11.705361; this version posted February 14, 2026. The copyright holder has placed this preprint (which was not certified by peer review) in the Public Domain. It is no longer restricted by copyright. Anyone can legally share, reuse, remix, or adapt this material for any purpose without crediting the original authors.

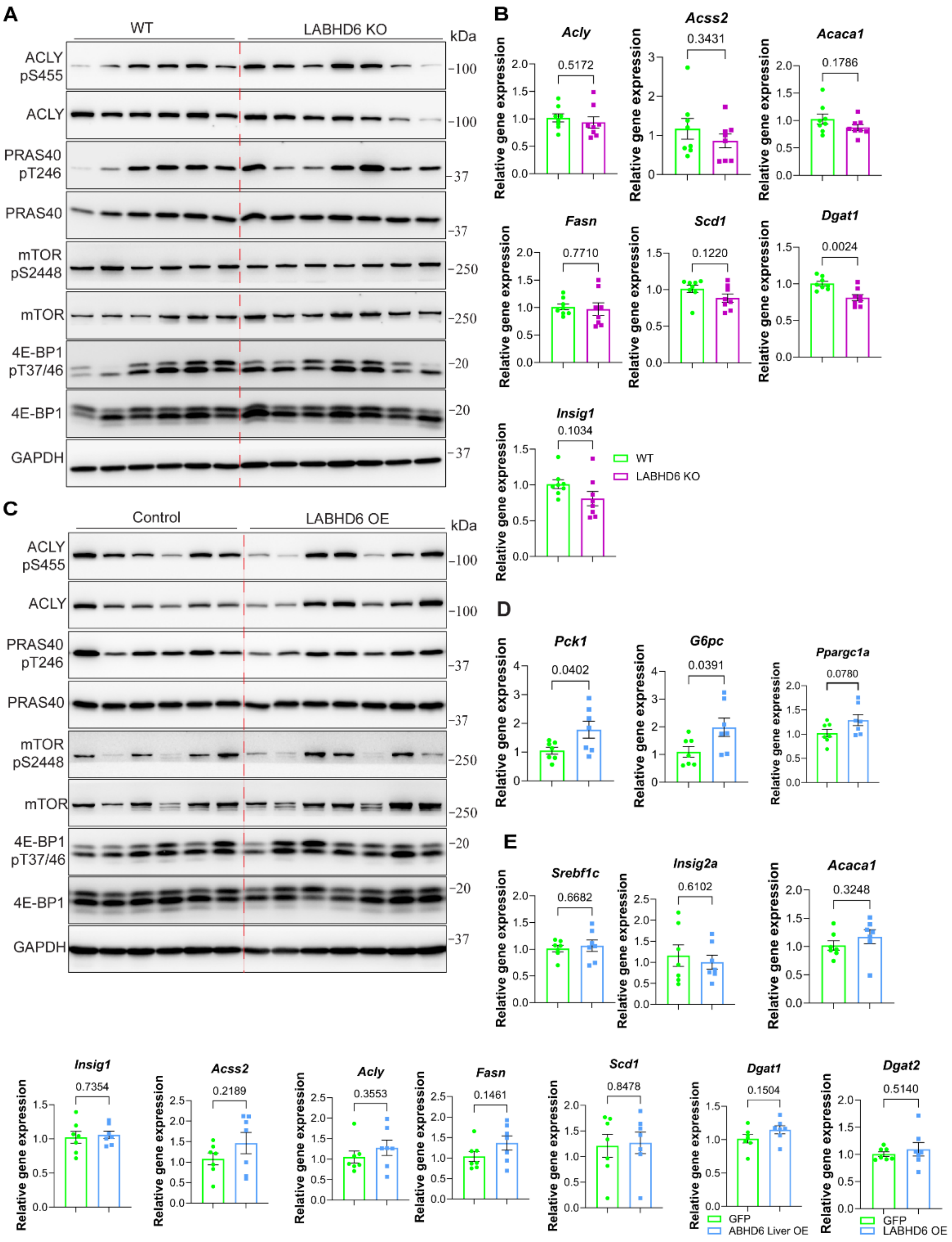

bioRxiv preprint doi: https://doi.org/10.64898/2026.02.11.705361; this version posted February 14, 2026. The copyright holder has placed this preprint (which was not certified by peer review) in the Public Domain. It is no longer restricted by copyright. Anyone can legally share, reuse, remix, or adapt this material for any purpose without crediting the original authors.

Sup Figure 6. ABHD6 manipulation only affects Akt-Foxo1/3a-mediated gluconeogenesis, without affecting Akt-mediated lipogenesis. Liver samples were obtained from LABHD6 KO and OE mice after HFD feeding. Western blot and qPCR analysis were performed to check the hepatic insulin signaling. (A) Hepatic insulin signaling in LABHD6 KO mice. (B) Gene expression of Acly, Acss2, Acaca1, Dgat1, and Insig1. (C) Hepatic insulin signaling in LABHD6 OE mice. (D) Gene expression of gluconeogenic genes, including *Pck1*, *G6pc* and *Ppargc1a* in the livers of Control and LABHD6 OE mice. (E) Gene expression lipogenic genes, including *Srebf1*, *Insig2a*, *Acaca1*, *Insig1*, *Acss2*, *Acly*, *Fasn*, *Scd1*, *Dgat1* and *Dgat2*., . Data are displayed as mean ± SEM and analyzed by Student's *t* test p-values of respective comparisons are provided.

bioRxiv preprint doi: https://doi.org/10.64898/2026.02.11.705361; this version posted February 14, 2026. The copyright holder has placed this preprint (which was not certified by peer review) in the Public Domain. It is no longer restricted by copyright. Anyone can legally share, reuse, remix, or adapt this material for any purpose without crediting the original authors.

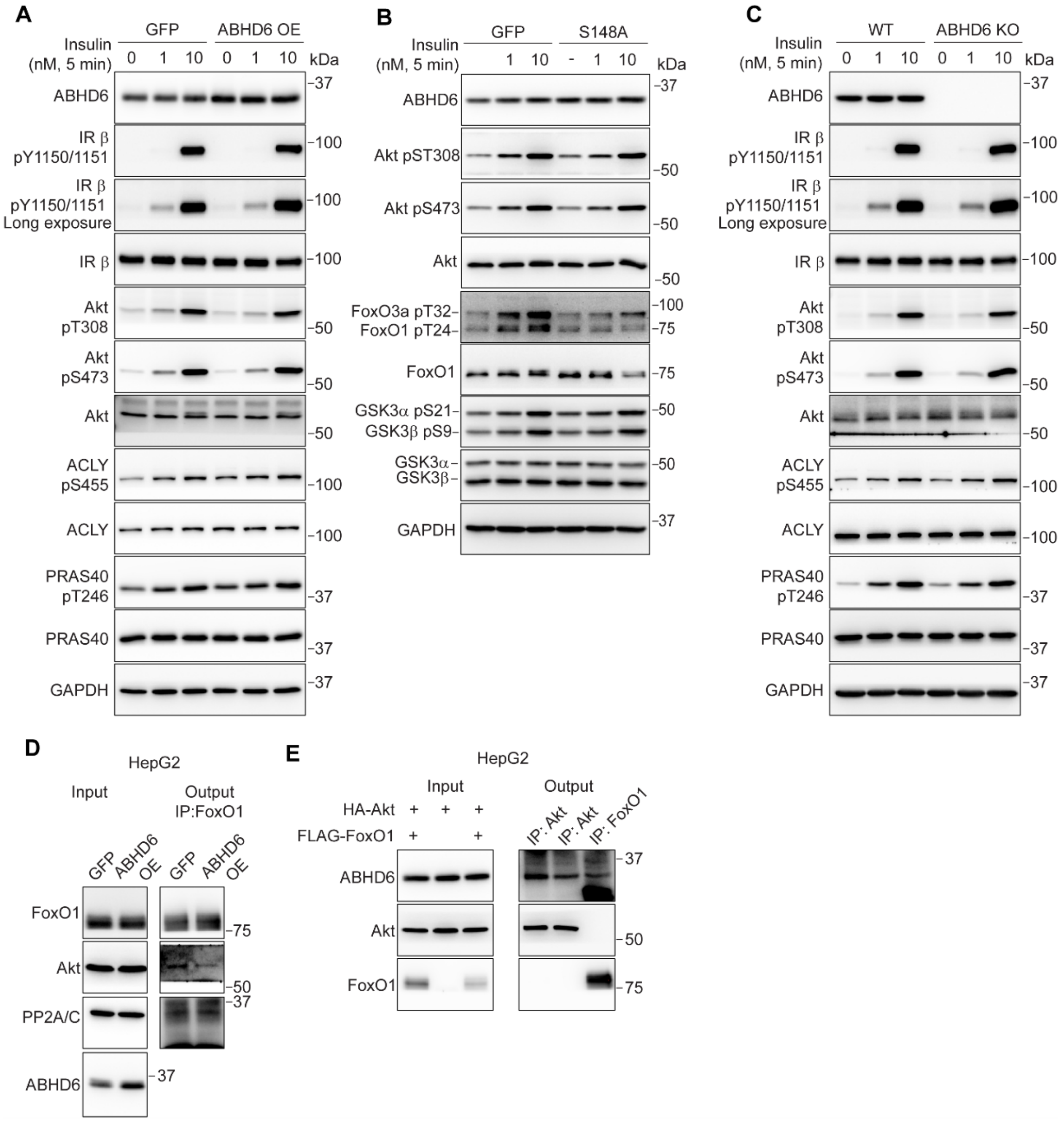

Sup Figure 7. ABHD6 regulates Akt-Foxo1/3a phosphorylation and interaction, leading to altered gluconeogenesis. (A) Effects of ABHD6 overexpression on hepatic insulin signaling.(B) Effects of ABHD6 S148A overexpression on hepatic insulin signaling. (C) Effects of ABHD6 knockout on hepatic insulin signaling. (D) FoxO1 interaction with PP2A/C in ABHD6 OE HepG2 cells. HepG2 cells transduced with AAVDJ CAG-Abhd6

bioRxiv preprint doi: https://doi.org/10.64898/2026.02.11.705361; this version posted February 14, 2026. The copyright holder has placed this preprint (which was not certified by peer review) in the Public Domain. It is no longer restricted by copyright. Anyone can legally share, reuse, remix, or adapt this material for any purpose without crediting the original authors.

(ABHD6 OE) or CAG-eGFP (GFP) at MOI of 1000. 48 hours post-transfection/transduction, FoxO1 co-immunoprecipitation was performed. (E) ABHD6 interaction with Akt2 or FoxO1 in HepG2 cells. HepG2 cells were transfected with 1 μg HA-Akt2 and/or FLAG-FoxO1 plasmids and transduced with AAVDJ CAG-Abhd6 at MOI of 1000. 48 hours post-transfection/transduction, Akt2 and FoxO1 co-immunoprecipitation was performed.

bioRxiv preprint doi: https://doi.org/10.64898/2026.02.11.705361; this version posted February 14, 2026. The copyright holder has placed this preprint (which was not certified by peer review) in the Public Domain. It is no longer restricted by copyright. Anyone can legally share, reuse, remix, or adapt this material for any purpose without crediting the original authors.

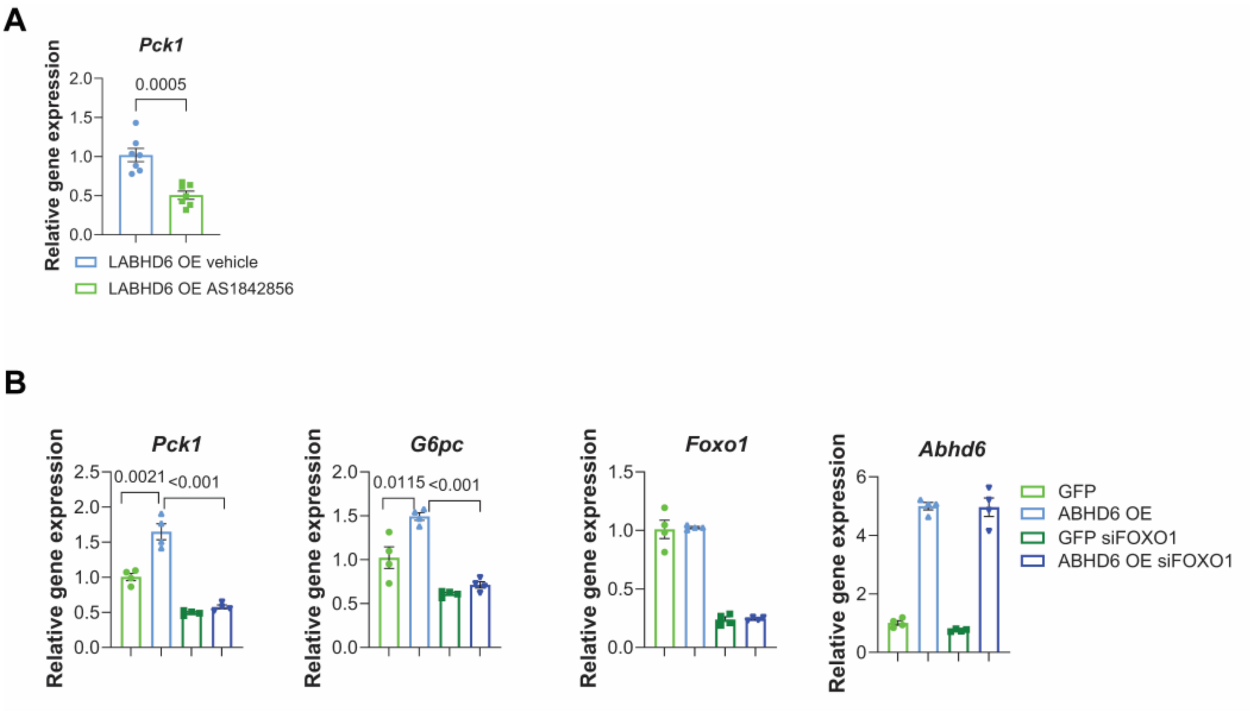

Sup Figure 8. ABHD6 regulates insulin sensitivity in a FoxO1-dependent manner.

(A) 8-week-old male wildtype mice were injected with 1×10^{11} genome copies TBG-Abhd6 (LABHD6 OE). One week post-injection, the mice were fed a high-fat diet (HFD) for 12 weeks. AS1842856 was administered orally at 30 mg/kg, three times over two days (see Methods for experimental details). Gene expression of *Pck1* in the livers. (B) Foxo1 knockdown completely abolishes the effects of ABHD6 overexpression on gluconeogenic gene expression in primary hepatocyte. Data are displayed as mean ± SEM and analyzed by Student's t test. p-values of respective comparisons are provided.

bioRxiv preprint doi: https://doi.org/10.64898/2026.02.11.705361; this version posted February 14, 2026. The copyright holder has placed this preprint (which was not certified by peer review) in the Public Domain. It is no longer restricted by copyright. Anyone can legally share, reuse, remix, or adapt this material for any purpose without crediting the original authors.

Supplemental Table 1 Primers used in the study

**Primers**	**Sequence (5'-3')**	**Gene**	**Species**
mPck-1-F	CCGGAGTATATCCACATCTG	Pck1	mouse
mPck-1-R	GGGTGATGATGACTGTCTTG	Pck1	mouse
mG6p-F	GTGGGCATCAATCTCCTCTG	G6pc1	mouse
mG6p-R	CGGAGCTGTTGCTGTAGTAG	G6pc1	mouse
mPgc1a-F	TATGGAGTGACATAGAGTGTGCT	Ppargc1a	mouse
mPgc1a-R	CCACTTCAATCCACCCAGAAAG	Ppargc1a	mouse
mAbhd6-F	CATTCCAATCCTGGCATTTGTTG	Abhd6	mouse
mAbhd6-R	ATGGTGTGCGTAGCGAACTT	Abhd6	mouse
mInsig1-F	CACGACCACGTCTGGAACTAT	Insig1	mouse
mInsig1-R	TGAGAAGAGCACTAGGCTCCG	Insig1	mouse
mInsig2a-F	CCCTCAATGGTACTGAAGCATT	Insig2a	mouse
mInsig2a-R	TGTGAACTCAAGCAGACCAATG	Insig2a	mouse
mSrebf1-F	TCAGCAGCCCCTAGAACAAA	Srebf1	mouse
mSreb1-R	CTGATGCCTGCAGTCTTCAC	Srebf1	mouse
mAcss2-F	AAACACGCTCAGGGAAAATCA	Acss2	mouse
mAcss2-R	ACCGTAGATGTATCCCCCAGG	Acss2	mouse
mAcly-F	ACCCTTTCACTGGGGATCACA	Acly	mouse
mAcly-R	GACAGGGATCAGGATTTCCTTG	Acly	mouse
mAcc1-F	GCCTCTTCCTGACAAACGAG	Acaca	mouse
mAcc1-R	TGACTGCCGAAACATCTCTG	Acaca	mouse

bioRxiv preprint doi: https://doi.org/10.64898/2026.02.11.705361; this version posted February 14, 2026. The copyright holder has placed this preprint (which was not certified by peer review) in the Public Domain. It is no longer restricted by copyright. Anyone can legally share, reuse, remix, or adapt this material for any purpose without crediting the original authors.

mScd1-F	ATGTCTGACCTGAAAGCCGA	Scd1	mouse
mScd1-R	GAAGGTGCTAACGAACAGGC	Scd1	mouse
mFasn-F	GCTTCGCCAACTCTACCATG	Fasn	mouse
mFasn-R	CCATCGCTTCCAGGACAATG	Fasn	mouse
mDgat1-F	TGTGTGGTGATGCTGATCCT	Dgat1	mouse
mDgat1-R	TGCAATAATCACGCATGGGG	Dgat1	mouse
mDgat2-F	GCGCTACTTCCGAGACTACT	Dgat2	mouse
mDgat2-R	ATCCGGAAGTTACCAGCCAA	Dgat2	mouse
mTnfα-F	GATCGGTCCCCAAAGGGATG	Tnfα	mouse
mTnfα-R	GTTTGCTACGACGTGGGCT	Tnfα	mouse
mIl-1β-F	CCAAAAGATGAAGGGCTGCT	Il-1β	mouse
mIl-1β-R	TCATCAGGACAGCCCAGGTC	Il-1β	mouse
mIl-1a-F	AGGAGAGCCGGGTGACAGTA	Il-1α	mouse
mIl-1a-R	TCAGAATCTTCCCGTTGCTTG	Il-1α	mouse
mIL6-F	AGCCAGAGTCCTTCAGAGAGAT	Il6	mouse
mIL6-R	GAGAGCATTGGAAATTGGGGT	Il6	mouse
mIl-8-F	CTGGTCCATGCTCCTGCTG	IL8	mouse
mIl-8-R	GGACGGACGAAGATGCCTAG	IL8	mouse
mCxcl1-F	CAATGAGCTGCGCTGTCAGT	Cxcl1	mouse
mCxcl1-R	TTGAGGTGAATCCCAGCCAT	Cxcl1	mouse
mF4/80-F	TGTCCAGCAATGGACAAACC	Adgre1	mouse
mF4/80-R	TCCCAGTGGAAGAAGAGAAG	Adgre1	mouse

bioRxiv preprint doi: https://doi.org/10.64898/2026.02.11.705361; this version posted February 14, 2026. The copyright holder has placed this preprint (which was not certified by peer review) in the Public Domain. It is no longer restricted by copyright. Anyone can legally share, reuse, remix, or adapt this material for any purpose without crediting the original authors.

mCpt1a-F	TCTCCGCCTGAGCCATGAAG	Cpt1a	mouse
mCpt1a-R	CACCCACCACCACGATAAGC	Cpt1a	mouse
mMcad-F	TGCCCGCAAGTTTGCCAGAG	MCAD	mouse
mMcad-R	AAGGCCACCGCAACTTTCCG	MCAD	mouse
mPpara-F	CAGTCCATCGGTGAGGAGAG	PPARA	mouse
mPpara-R	GACTGAGGAAGGGCTGGAAG	PPARA	mouse
mTgfb-F	CTCCCGTGGCTTCTAGTGC	TGFB	mouse
mTgfb-R	GCCTTAGTTTGGACAGGATCTG	TGFB	mouse
mCcl2-F	CCTGCTGCTACTCATTCACCA	Ccl2/Mcp1	mouse
mCcl2-R	ATTCCTTCTTGGGGTCAGCA	Ccl2/Mcp1	mouse
mCcl7-F	GAGGATCTCTGCCACGCT TC	Ccl7	mouse
mCcl7-F	ACACCGACTACTGGTGATCC	Ccl7	mouse
mMmp2-F	CAAGTTCCCCGGCGATGTC	Mmp2	mouse
mMmp2-R	TTCTGGTCAAGGTCACCTGTC	Mmp2	mouse
mCxcl10-F	ATGACGGGCCAGTGAGAATG	Cxcl10	mouse
mCxcl10-R	TCAACACGTGGGCAGGATAG	Cxcl10	mouse
mActa2-F	GTCCCAGACATCAGGGAGTAA	Acta2	mouse
mActa2-R	TCGGATACTTCAGCGTCAGGA	Acta2	mouse
mCol1a1-F	GCTCCTCTTAGGGGCCACT	Col1a1	mouse
mCol1a1-R	CCACGTCTCACCATTGGGG	Col1a1	mouse
mCol3a1-F	GGCACAGCAGTCCAACGTAG	Col3a1	mouse
mCol3a1-R	CGCAAAGGACAGATCCTGAG	Col3a1	mouse
m16S-F	GATTTGCTGGTGTGGATATT	16S	mouse

bioRxiv preprint doi: https://doi.org/10.64898/2026.02.11.705361; this version posted February 14, 2026. The copyright holder has placed this preprint (which was not certified by peer review) in the Public Domain. It is no longer restricted by copyright. Anyone can legally share, reuse, remix, or adapt this material for any purpose without crediting the original authors.

m16S-R	TCTTTGATCTCCTTCTTGGA	16S	mouse

bioRxiv preprint doi: https://doi.org/10.64898/2026.02.11.705361; this version posted February 14, 2026. The copyright holder has placed this preprint (which was not certified by peer review) in the Public Domain. It is no longer restricted by copyright. Anyone can legally share, reuse, remix, or adapt this material for any purpose without crediting the original authors.

Supplemental Table 2 Antibodies used in the study

**Antibody**	**Source**	**Identifier**
ABHD6	Cell signaling technology	Cat#97573;<br>RRID: AB_2800281
FoxO1	Cell signaling technology	Cat#2880;<br>RRID:AB_2106495
phospho-FoxO1 (Thr24)/FoxO3a (Thr32)	Cell signaling technology	Cat#9464;<br>RRID:AB_329842
phospho-IGF-I Receptor β (Tyr1135/1136)/Insulin Receptor β (Tyr1150/1151)	Cell signaling technology	Cat#3024;<br>RRID:AB_331253
Insulin Receptor β	Cell signaling technology	Cat#3025;<br>RRID:AB_2280448
phospho-Akt (Ser473)	Cell signaling technology	Cat#4060;<br>RRID:AB_2315049
phospho-Akt (Thr308)	Cell signaling technology	Cat#13038;<br>RRID:AB_2629447
Akt (pan)	Cell signaling technology	Cat#4691;<br>RRID:AB_915783
phospho-GSK-3α/β (Ser21/9)	Cell signaling technology	Car#8566;<br>RRID:AB_10860069
GSK-3α/β	Cell signaling technology	Cat#5676;

bioRxiv preprint doi: https://doi.org/10.64898/2026.02.11.705361; this version posted February 14, 2026. The copyright holder has placed this preprint (which was not certified by peer review) in the Public Domain. It is no longer restricted by copyright. Anyone can legally share, reuse, remix, or adapt this material for any purpose without crediting the original authors.

		RRID:AB_10547140
phospho-ATP-Citrate Lyase (Ser455)	Cell signaling technology	Cat#4331; RRID:AB_2257987
ATP-Citrate Lyase	Cell signaling technology	Cat#4332; RRID:AB_2223744
phospho-PRAS40 (Thr246)	Cell signaling technology	Cat#13175; RRID:AB_2798140
PRAS40	Cell signaling technology	Cat#2691; RRID:AB_2225033
phospho-mTOR (Ser2448)	Cell signaling technology	Cat#5536; RRID:AB_10691552
mTOR	Cell signaling technology	Cat#2972; RRID:AB_330978
phospho-4E-BP1 (Thr37/46)	Cell signaling technology	Cat#2855; RRID:AB_560835
4E-BP1	Cell signaling technology	Cat#9644; RRID:AB_2097841
LSD1	Cell signaling technology	Cat#2184; RRID:AB_2070132
β-Actin	Abclonal	Cat#AC004; RRID:AB_2737399
GAPDH	Abclonal	Cat#AC002; RRID:AB_2736879
